# Impella-Supported Optical Coherence Tomography-Guided Aggressive Rotational Atherectomy for Heavily Calcified Lesions in Left Main Trunk Bifurcation in a Patient with Severe Left Ventricular Systolic Dysfunction

**DOI:** 10.1155/2023/6894610

**Published:** 2023-08-14

**Authors:** Masahiro Koide, Kento Fukui, Hikaru Sugimoto, Yuki Takeda, Koji Sogabe, Yukinori Kato, Hiroki Kitajima, Satoshi Akabame

**Affiliations:** Department of Cardiovascular Medicine, Kyoto Okamoto Memorial Hospital, Kyoto, Japan

## Abstract

The Impella, a percutaneous left ventricular assist device, has been reported to minimize the risk of hemodynamic compromise and improve clinical outcomes during percutaneous coronary intervention (PCI) in complex high-risk indicated patients (CHIPs). Optical coherence tomography (OCT) provides information on calcified plaque thickness, which is helpful in determining the indication and endpoint of atherectomy during PCI for calcified lesions. However, there are few reports on OCT-guided aggressive rotational atherectomy with Impella assistance in CHIPs. A 71-year-old man on dialysis for end-stage renal failure was admitted for congestive heart failure. Transthoracic echocardiography revealed severe left ventricular systolic dysfunction, and coronary angiography performed after improvement of heart failure showed severe stenosis with heavily calcified lesions in the left main trunk (LMT) bifurcation and right coronary artery. The patient refused coronary artery bypass surgery and was revascularized using PCI. PCI was started with prophylactic Impella CP insertion because of the high risk of hemodynamic collapse. After OCT-guided rotational atherectomy with 1.5- and 2.0-mm burr toward the left anterior descending artery and left circumflex artery, respectively, double-kissing culotte stenting was performed in the LMT, and good dilation was obtained. Impella CP was removed immediately after PCI without hemodynamic compromise, and the procedure was completed.

## 1. Introduction

The use of Impella, a percutaneous left ventricular assist device, has been demonstrated in multiple clinical trials to minimize the risk of hemodynamic compromise, and enable complete revascularization, resulting in the improvement of clinical outcomes during percutaneous coronary intervention (PCI) in complex high-risk indicated patients (CHIPs), including those with severely depressed left ventricular ejection fraction (LVEF) [1, 2]. Optical coherence tomography (OCT) is a high-resolution intravascular imaging device that provides information on the thickness of calcified coronary plaques, which is useful in determining the indication and endpoint of atherectomy during PCI for calcified lesions [3, 4]. However, there have been few reports of PCI with OCT-guided aggressive rotational atherectomy in CHIPs with highly calcified lesions.

## 2. Case Report

**Figure 1:**
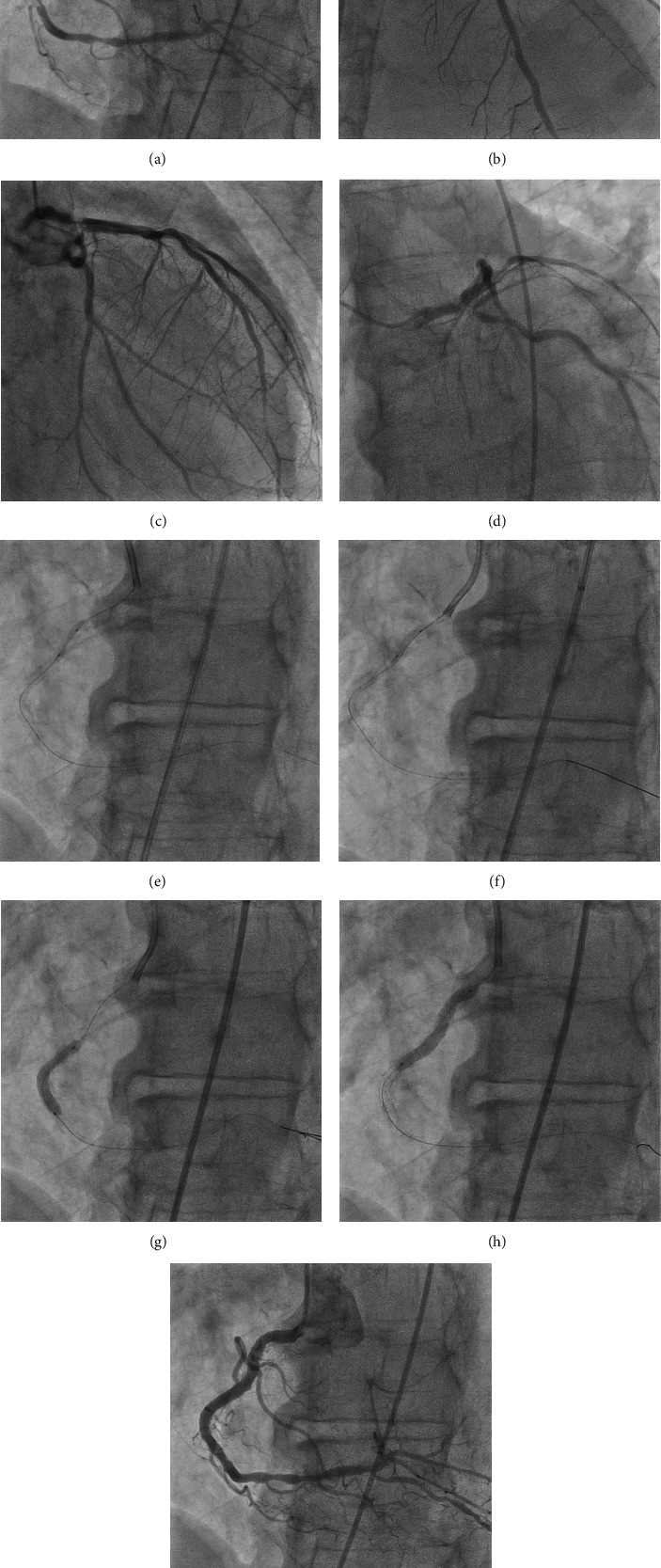
Coronary angiography and PCI of RCA. Coronary angiography revealed severe stenosis with heavy calcification in the proximal and middle portions of the RCA (a) and in the bifurcation of the LMT (b–d). The calcified lesion could not be dilated with a 2.0-mm semi-compliant balloon (e) and was successfully dilated with a 2.0-mm Scoreflex™ scoring balloon (f), followed by implanting with 3.5-mm × 28-mm and 4.0-mm × 28-mm sirolimus-eluting stent of Ultimaster Tansei™ (g and h), successfully resulting in good dilatation (i).

A 71-year-old man on hemodialysis for end-stage renal failure was admitted to our hospital with dyspnea and chest discomfort. Electrocardiography (ECG) revealed ST-segment depression in leads V5 and V6, and a chest X-ray revealed severe pulmonary congestion, leading to a diagnosis of congestive heart failure and emergency admission. Transthoracic echocardiography revealed diffuse severe left ventricular systolic dysfunction with an LVEF of 32%. Heart failure improved significantly by controlling fluid volume with hemodialysis. Coronary angiography was performed to investigate the cause of the left ventricular dysfunction, which revealed severe stenosis with heavy calcifications in the proximal and middle portions of the right coronary artery (RCA) and the bifurcation of the left coronary main trunk (LMT; Figures 1(a), 1(b), 1(c), and 1(d)). We strongly recommended that he should undergo coronary artery bypass graft surgery; however, he refused to do so. Therefore, we decided to perform complete revascularization via a PCI. First, PCI was performed on the RCA (Figures 1(e), 1(f), 1(g), 1(h), and 1(i)) via the left femoral approach. A 2.0-mm semi-compliant balloon failed to dilate the calcified lesion (Figure 1(e)), but a 2.0-mm scoring balloon (Scoreflex™, OrbusNeich, Hong Kong, China) successfully dilated the stenotic lesion by pressurizing it to 30 atm (Figure 1(f)), followed by dilation using a 3.5-mm high-pressure balloon and implantation with 3.5-mm × 28-mm and 4.0-mm × 28-mm sirolimus-eluting stent of Ultimaster Tansei™ (Terumo Corp., Tokyo, Japan; Figures 1(g) and 1(h)), successfully resulting in adequate dilatation without hemodynamic compromise (Figure 1(i)). For the heavily calcified lesion in the LMT, there was a concern that it would result in stent under-expansion and an increased risk of future restenosis without rotational atherectomy. However, given the patient's limited cardiac function, rotational atherectomy without mechanical circulatory support would likely result in hemodynamic compromise. Therefore, we decided to perform PCI on the LMT lesions while providing circulatory support with Impella CP™ (Abiomed Inc., Danvers, MA, USA).

**Figure 2:**
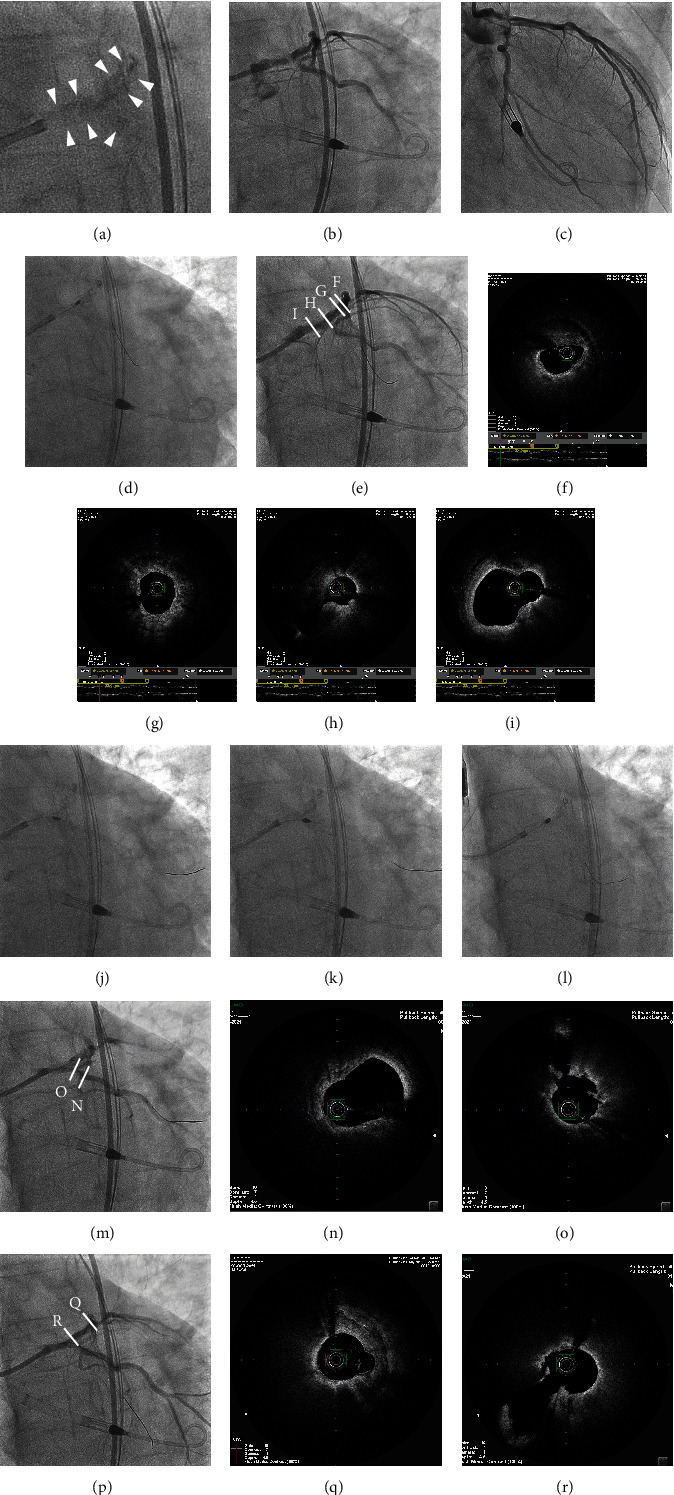
Impella-supported OCT-guided rotational atherectomy for LMT bifurcation lesion. The heavy calcification at the bifurcation of the left main coronary artery was visible on fluoroscopy (a). Coronary angiography before PCI (b and c). Rotational atherectomy with a 1.5-mm RotaPRO™ was performed from the LMT to the proximal LAD artery (d) and proximal left circumflex artery (LCx) (j). OFDI images pulled back from LAD (e–i). Then, additional atherectomy was performed from the LMT to the proximal LCx (k) and LAD (l) using a 2.0-mm RotaPRO™, and the lesions were again observed by OFDI, confirming that the calcified plaque had sufficiently reduced in volume (m–r).

**Figure 3:**
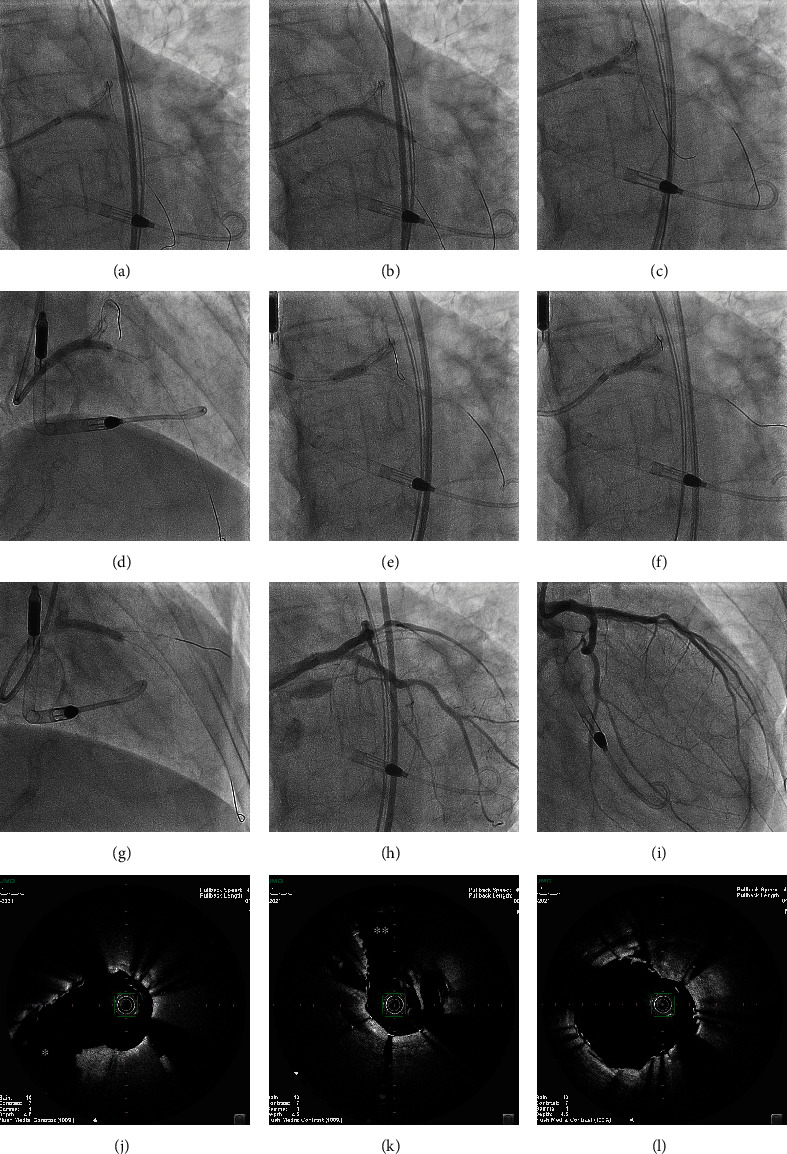
Double-kissing culotte stenting for LMT bifurcation lesion. After dilation with a 3.0-mm high-pressure balloon from the proximal LCx to the LMT (a), a 3.0-mm × 28-mm Ultimaster Tansei™ stent was implanted from the LMT to the proximal LCx (b). KBI was performed with 3.5- and 3.0-mm balloons (c). A 3.5-mm × 24-mm Ultimaster Tansei™ stent was implanted from the proximal LAD to the LMT (d), followed by a proximal optimization technique with a 4.5-mm balloon in the LMT (e), and KBI was performed with 3.5- and 3.0-mm balloons (f). A 3.5-mm × 18-mm stent of Ultimaster Tansei™ was implanted in the distal portion of proximal LAD (g). Coronary angiography (h and i) and OFDI images (j–l) confirmed good flow and good dilatation of lesions.

An 8-Fr sheath was inserted through the right femoral artery, and after suturing the vessel wall at the puncture site using a 6-Fr Perclose Proglide™ (Abbott Vascular Inc., Temecula, CA, USA; “preclose technique”), the sheath was replaced with a 14-Fr sheath to start pumping the Impella CP™. After the start of Impella CP™, the level of assistance was set at P-8, and the flow rate was 3.3 L/minutes. The hemostatic valve of the Impella insertion sheath was punctured, and a 7-Fr sheath was inserted using a method previously identified as a single-access technique [5]. A 7-Fr SPB 3.5 with a side hole (Asahi Intecc Corp., Nagoya, Japan) guiding catheter was inserted into the left coronary artery. The heavy calcification at the bifurcation of the left main coronary artery was visible on fluoroscopy (Figure 2(a)). Because the optical frequency domain imaging (OFDI) catheter could not pass through the lesions, rotational atherectomy with a 1.5-mm RotaPRO™ (Boston Scientific Corp., Marlborough, MA, USA) with RotaWire Extrasupport™ (Boston Scientific Corp.) at 200,000 rpm was performed from the LMT to the proximal left anterior descending (LAD) artery and proximal left circumflex artery (LCx; Figures 2(d) and 2(g)), and then each lesion was observed via OFDI (Terumo Corp.). The calcified plaques were found to have been partially ablated. However, some remained as entire circumferential calcified plaques with a maximum thickness of 1.5 mm (Figures 2(f), 2(g), 2(g), 2(h), and 2(i)). Next, additional atherectomy was performed from the LMT to the proximal LCx and LAD using a 2.0-mm RotaPRO™ (Figures 2(j) and 2(m)). The total ablation time of the rotational atherectomy was 345 seconds. Despite aggressive rotational atherectomy, systolic blood pressure stabilized around 120 mmHg intraoperatively, and there was no decrease in coronary blood flow or ST-segment elevation on the ECG. The lesions were again observed via OFDI, confirming that the calcified plaque had sufficiently reduced in volume (Figures 2(m), 2(n), 2(o), 2(p), 2(q), and 2(r)). After dilation using a 3.0-mm high-pressure balloon from the proximal LCx to the LMT (Figure 3(a)), a 3.0-mm × 28-mm Ultimaster Tansei™ stent was implanted from the LMT to the proximal LCx (Figure 3(b)). Then, after recrossing the guidewire into the LAD, kissing balloon inflation (KBI) was performed using 3.5- and 3.0-mm balloons (Figure 3(c)). Then, a 3.5-mm × 24-mm Ultimaster Tansei™ stent was implanted from the LMT to the proximal LAD (Figure 3(d)), followed by a proximal optimization technique using a 4.5-mm balloon in the LMT (Figure 3(e)), recrossing the guidewire to the LCx. Again, KBI was performed using 3.5- and 3.0-mm balloons (Figure 3(f)), a procedure previously reported as the double-kissing culotte technique. Thereafter, a 3.5-mm × 18-mm stent of Ultimaster Tansei™ was implanted in the distal portion of the proximal LAD (Figure 3(g)). Coronary angiography and OFDI images confirmed good flow and good dilatation of lesions (Figures 3(h), 3(i), 3(j), 3(k), and 3(l)). The Impella CP™ and 14-Fr sheath were removed immediately after PCI, and hemostasis was completed using two types of hemostatic devices, Perclose Proglide™ (Abbott Vascular) and Angio-Seal VIP 8F (Terumo Corp.). Despite aggressive debulking of heavily calcified bifurcated lesions in the LMT in a patient with severely impaired left ventricular systolic function, no intraoperative hemodynamic or respiratory compromise was observed, and neither oxygen nor catecholamine treatment was needed. The day after PCI, the CPK level was 205 U/L, within the normal range. He was discharged from the hospital four days after PCI without any complications. Thirteen months later, transthoracic echocardiography revealed a marked improvement in LVEF (which increased to 60%) with no recurrence of chest pain or cardiovascular events.

## 3. Discussion

Rotational atherectomy during PCI without mechanical circulatory support in patients with severe left ventricular systolic dysfunction is associated with a high risk of the no-reflow phenomenon and critical hemodynamic collapse [6]. Therefore, the efficacy of Impella-supported PCI for such CHIP cases has been reported in several clinical studies [1, 2]. Furthermore, more remarkable LVEF improvement has been reported with Impella-assisted PCI in patients with severe left ventricular systolic dysfunction, as shown in the present case [7]. Despite the demonstrated efficacy of Impella in PCI for these CHIP cases, the prophylactic use of Impella for these cases is unfortunately not covered by insurance in some regions, including Japan, and further indication expansion is expected.

Intra-aortic balloon counterpulsation (IABP) is the most common mechanical circulatory support device used during PCI in patients with unstable hemodynamics. IABP has been reported to increase diastolic blood pressure in the aorta and non-stenotic coronary arteries but not in significantly stenotic coronary arteries [8]. On the other hand, it has been reported that Impella not only has a more potent circulatory support effect than IABP but also improves coronary perfusion by increasing coronary artery pressure distal to the severe stenosis and decreasing left ventricular end-diastolic pressure [9]. As a result of this improvement in coronary circulation, Impella may reduce the risk of the no-reflow phenomenon during rotational atherectomy, which would improve postoperative cardiac function. On the other hand, another powerful circulatory assist device, venoarterial extracorporeal membrane oxygenation (VA-ECMO), increases the myocardial oxygen demand by increasing left ventricular afterload and wall stress; and as a result, observational studies have reported worse outcomes with circulatory assistance with VA-ECMO compared with impellers in PCI in CHIPs [10].

OCT is a high-resolution intravascular imaging device [3, 4]. In PCI for usual cases, OCT-guided PCI has proven to be as safe and effective as intravascular ultrasound (IVUS)-guided PCI. Compared with IVUS-guidance, OCT-guided PCI provides about ten times higher resolution images and information on the calcified plaque thickness, which helps determine the indication for atherectomy and endpoints [3, 4]. Because rotational atherectomy in patients with severe left ventricular systolic dysfunction is associated with a high risk of the no-reflow phenomenon [6], more careful consideration should be given to determine the indications and endpoints of atherectomy. Therefore, OCT-guided PCI might be more beneficial for such patients. However, there are only a few reports on OCT-guided PCI for CHIP patients with highly calcified lesions as in the present case report.

Even if IVUS-guided PCI had been performed in this case, the therapeutic strategy might not have differed significantly from OCT-guided. This is because the PCI, in this case, was performed with robust circulatory support by Impella CP™, which allowed aggressive debulking with less risk of no-reflow or hemodynamic collapse by upgrading the size of the rotational atherectomy. On the other hand, when PCI is performed without mechanical circulatory assistance in patients with impaired cardiac function, information on calcified plaque thickness obtained by OCT could be more important in determining the endpoint of rotational atherectomy.

Here, we describe a case in which an Impella-supported OCT-guided aggressive rotational atherectomy was successfully performed for a left main bifurcation lesion with a heavily calcified lesion in a patient with severe left ventricular systolic dysfunction.

## Data Availability

No underlying data was collected or produced in this study.
